# Chalcone flavokawain A attenuates TGF‐β1‐induced fibrotic pathology via inhibition of ROS/Smad3 signaling pathways and induction of Nrf2/ARE‐mediated antioxidant genes in vascular smooth muscle cells

**DOI:** 10.1111/jcmm.13973

**Published:** 2018-12-13

**Authors:** You‐Cheng Hseu, Ting‐Yu Yang, Mei‐Ling Li, Peramaiyan Rajendran, Dony Chacko Mathew, Chia‐Hsuan Tsai, Ruei‐Wan Lin, Chuan‐Chen Lee, Hsin‐Ling Yang

**Affiliations:** ^1^ Department of Cosmeceutics College of Biopharmaceutical and Food Sciences China Medical University Taichung Taiwan; ^2^ Department of Health and Nutrition Biotechnology Asia University Taichung Taiwan; ^3^ Chinese Medicine Research Center China Medical University Taichung Taiwan; ^4^ Research Center of Chinese Herbal Medicine China Medical University Taichung Taiwan; ^5^ Institute of Nutrition College of Biopharmaceutical and Food Sciences China Medical University Taichung Taiwan

**Keywords:** fibrosis, flavokawain A, Nrf2, ROS, Smad3, smooth muscle cells, TGF‐β1p

## Abstract

TGF‐β1 plays a crucial role in the pathogenesis of vascular fibrotic diseases. Chalcones are reportedly cancer chemo‐preventive food components that are rich in fruits and vegetables. In this study, flavokawain A (FKA, 2‐30 μM), a naturally occurring chalcone in kava extracts, was evaluated for its anti‐fibrotic and antioxidant properties in TGF‐β1‐stimulated vascular smooth muscle (A7r5) cells, as well as its underlying molecular mechanism of action. Immunofluorescence data showed down‐regulated F‐actin expression with FKA treatment in TGF‐β1‐stimulated A7r5 cells. Western blotting demonstrated that FKA treatment suppressed the expression of α‐SMA and fibronectin proteins under TGF‐β1 stimulation. Findings from wound‐healing and invasion experiments showed that FKA inhibits TGF‐β1‐mediated migration and invasion. Western blotting demonstrated that treatment with FKA down‐regulated MMP‐9 and MMP‐2 and up‐regulated TIMP‐1 expression. Further evidence showed that FKA decreased TGF‐β1‐mediated phosphorylation and the transcriptional activity of Smad3. TGF‐β1‐induced excessive ROS production was remarkably reversed by FKA treatment in A7r5 cells, and inhibition by FKA or *N*‐acetylcysteine (NAC) substantially diminished TGF‐β1‐induced p‐Smad3 activation and wound‐healing migration. Interestingly, FKA‐mediated antioxidant properties were associated with increased nuclear translocation of Nrf2 and elevated antioxidant response element (ARE) luciferase activity. Activation of Nrf2/ARE signaling was accompanied by the induction of HO‐1, NQO‐1 and γ‐GCLC genes in FKA‐treated A7r5 cells. Notably, silencing of Nrf2 (siRNA transfection) significantly diminished the FKA‐mediated antioxidant effects, indicating that FKA may inhibit TGF‐β1‐induced fibrosis through suppressing ROS generation in A7r5 cells. Our results suggested that anti‐fibrotic and antioxidant activities of the chalcone flavokawain A may contribute to the development of food‐based chemo‐preventive drugs for fibrotic diseases.

## 1 INTRODUCTION

Fibrosis can be described as a non‐physiological scarring process in response to chronic diseases, wherein excessive extracellular matrix (ECM) deposition contributes to irreversible tissue damage and the malfunction of vital organs, including the liver, heart, lung, kidney and skin.^1^, ^2^ Various chemokines and growth factors are primarily involved in the onset and progression of fibrotic pathology; the cytokine transforming growth factor‐β (TGF‐β) is a crucial regulator of all types of fibrosis.^3^ TGF‐β family members are multifunctional proteins that regulate diverse cellular functions, including cell division, migration, invasion, adhesion, promotion of ECM production, tissue homeostasis and embryogenesis.^4^, ^5^, ^6^ Of the 3 isoforms (TGF‐β1, 2 and 3), TGF‐β1 is the most important isoform for the cardiovascular system and is present in vascular smooth muscle cells, endothelial cells, myofibroblasts, macrophages and other hematopoietic cells.^6^ Perturbation of TGF‐β1 signaling pathways has been implicated in diverse human diseases, including fibrotic, autoimmune and cardiovascular diseases.^4^, ^5^ In particular, TGF‐β1 exerts pleiotropic effects on cardiovascular cells and promotes vascular fibrotic diseases, including hypertension, atherosclerosis, cardiac hypertrophy and heart failure.^4^, ^6^ TGF‐β1‐induced vascular fibrosis is characterized by cytoskeletal rearrangements and alterations in F‐actin assembly.^7^, ^8^ The increased ED‐A form of fibronectin (ECM proteins) by TGF‐β1 is required for the enhancement of α‐smooth muscle actin (α‐SMA) and collagen during fibrotic changes and wound healing.^9^, ^10^ TGF‐β1 is known to activate several ECM components, including fibronectin, an essential protein for enhancement of α‐SMA involved in fibrosis.^9^ Myofibroblasts, which express α‐smooth muscle actin (α‐SMA) and show an enormous capacity for producing ECM and collagen (types I and III), also inhibit the activity of matrix metalloproteinases (MMPs) and are known to be the main effector cells responsible for cardiac fibrosis.^11^, ^12^ At the molecular level, TGF‐β predominantly transmits signals through cytoplasmic proteins called Smads, which play a key role in vascular fibrosis. In VSMCs, TGF‐β1 increases the phosphorylation of receptor‐associated Smads (Smad2 and Smad3), which form heterodimers with Smad4. This complex translocates into the nucleus and triggers the transcription of genes, including fibronectin and type 1 collagen, that are involved in vascular fibrosis.^13^, ^14^ TGF‐β1 is extensively involved in the development of fibrosis in various organs by disturbing the homeostatic microenvironment and promoting cell migration, invasion or hyperplastic changes.^15^, ^16^ MMPs, a family of proteolytic enzymes, degrade the ECM components and play an important role in cell migration and invasion. TIMP (tissue inhibitor of MMP) reduces excessive ECM degradation, and an imbalance in the MMPs to TIMPs ratio underlies the pathogenesis of vascular fibrosis.^17^ The fibrotic events associated with TGF‐β1 are coincident with the induction of ROS‐producing enzymes and/or reduction of ROS scavenging enzymes.^18^, ^19^ In these circumstances, the redox‐sensitive protein nuclear factor E2‐related factor 2 (Nrf2) is reported to be involved in the dynamics of fibrogenesis.^18^ In the presence of ROS, Nrf2 disassociates from its bound form (Nrf2‐Keap1 complex), translocates to the nucleus and binds to a small Maf protein/ARE to induce the expression of a battery of antioxidant genes, including γ‐GCLC, HO‐1 and NQO‐1.^20^, ^21^, ^22^ Supplementation of antioxidants appears to modulate the degree of plasticity and severity of fibrosis.^18^ Therefore, inhibition of TGF‐β1 signaling or TGF‐β1‐mediated ROS/Smad3 signaling might be a potential therapeutic strategy to treat vascular fibrosis.^18^

Flavokawain A (FKA) is a naturally occurring chalcone, extracted from *Piper methysticum Forst*, commonly known as kava‐kava. It has been reported that the age‐standardized incidence of cancer in three kava‐drinking Pacific counties (Fiji, Vanuatu and Samoa) is significantly lower than in their neighboring counties (Australia and New Zealand).^23^ The therapeutic actions of kava extracts have been attributed to the presence of various secondary metabolites, including chalcones (flavokawains), alkaloids and several unique lactones (kavalactones).^24^ To date, three types of flavokawains (FKA, FKB and FKC) have been identified, and FKA is the predominant chalcone, constituting up to 0.46% of kava extracts.^25^ Consumption of kava extracts (kavalactones, FKB and contaminant hepatotoxins) has been reported to produce hepatotoxicity in humans and animals, but convincing evidence of kava‐induced hepatotoxicity has not yet been established.^26^ Previous studies have demonstrated that FKA preferably inhibits the growth of various cancer cells with no or minimal effect on the growth of several normal and cancer cells.^25^, ^27^, ^28^ Dietary feeding of FKA to mice resulted in no adverse effects on major organ functions, instead inducing phase II antioxidant enzymes in liver, lung, prostate and bladder tissues.^29^ Nevertheless, the effects of FKA on vascular muscle fibrosis and signaling molecules involved in fibrogenesis have not yet been investigated. We hypothesized that FKA could alleviate TGF‐β1‐mediated ROS/Smad3 signaling, thereby preventing fibrotic pathology in vascular smooth muscle cells. We determined the key molecular proteins demonstrating anti‐fibrotic effects in FKA and revealed the underlying induction of antioxidant Nrf2/ARE signaling pathways.

## 2 MATERIALS AND METHODS

### 2.1 Reagents and antibodies

Dulbecco's modified Eagle's medium (DMEM), fetal bovine serum (FBS), L‐glutamine and penicillin/streptomycin were obtained from GIBCO BRL/Invitrogen (Carlsbad, CA, USA). Flavokawain A (FKA) was purchased from LKT Laboratories, Inc. (St Paul, MN, USA). TGF‐β1 was purchased from Pepro TechInc. (Rocky Hill, NJ, USA). Antibodies against phospho‐Smad3, Smad3, phospho‐Smad2 and Smad2 were purchased from Cell Signaling Technology, Inc. (Danvers, MA, USA). Antibodies against fibronectin, MMP‐9, MMP‐2, TIMP‐1, F‐actin, Nrf2, NQO‐1, Keap‐1, histone and β‐actin were purchased from Santa Cruz Biotechnology, Inc. (Heidelberg, Germany). 4′,6‐Diamidino‐2‐phenylindole dihydrochloride (DAPI) was obtained from Calbiochem (La Jolla, CA, USA). 3‐(4, 5‐dimethylthiazol‐2‐yl)‐2,5‐diphenyltetrazolium bromide (MTT), dihydrofluorescein‐diacetate (DCFH_2_‐DA) and *N*‐acetyl‐cysteine (NAC) were purchased from Sigma‐Aldrich (St. Louis, MO, USA). Antibodies against HO‐1 and α‐SMA were purchased from Abcam (Cambridge, UK). All other chemicals were purchased from either Merck & Co., Inc. (Darmstadt, Germany) or Sigma‐Aldrich (St. Louis, MO, USA).

### 2.2 Cell culture

A rat aortic smooth muscle cell line (A7r5) was obtained from the American Type Culture Collection (ATCC, Manassas, VA, USA) and grown in DMEM medium supplemented with 10% fetal calf serum, 100 μg/mL penicillin and 1 μg/mL streptomycin, at 37°C with 5% CO_2_ in humidified conditions. Cultures were harvested and the cell number was determined by counting cell suspensions with a hemocytometer.

### 2.3 Sample treatment

For all TGF‐β1 stimulated experiments, the supernatant was removed after FKA supplementation for 2 h, the cells were washed with phosphate‐buffered saline (PBS) and the culture medium was replaced with new medium and then stimulated with or without TGF‐β1 (10 ng/mL) for 24 h.

### 2.4 MTT assay

Cell viability was determined by the MTT colorimetric assay. Cells (5 × 10^4^ cells/well in 24‐well plates) were treated with the indicated concentration of FKA alone for 24 h, or pre‐treated with FKA for 2 h and then stimulated with or without TGF‐β1 (10 ng/mL) for 24 h. MTT (0.5 mg/mL) in PBS was added to each well. After incubation at 37°C for 4 h, an equal volume of DMSO (400 μL) was added to dissolve the MTT formazan crystals and the absorbance was measured at 570 nm (A_570_) using an ELISA microplate reader (μ‐Quant, Winoosky, VT, USA). The percentage (%) of cell viability was calculated as: (A_570_ of treated cells/A_570_ of untreated cells) × 100.

### 2.5 Protein isolation and Western blot analysis

A7r5 cells were seeded in a 10‐cm dish at a density of 1 × 10^6^ cells/dish. Next, the cells were incubated with the indicated concentrations of FKA for 2 h, then stimulated with or without TGF‐β1 (10 ng/mL) for 24 h. Cells were detached and washed once in ice‐cold PBS and then re‐suspended in 100 μL lysis buffer containing 10 mM Tris‐HCl [pH 8], 0.32 M sucrose, 1% Triton X‐100, 5 mM EDTA, 2 mM dithiothreitol and 1 mM phenylmethyl sulfonyl fluoride. The suspension was kept on ice for 20 min and then centrifuged at 15,000 × *g* for 30 min at 4°C. Total protein content was determined using the Bio‐Rad protein assay reagent, with bovine serum albumin as a standard. Protein extracts were reconstituted in sample buffer (0.062 M Tris‐HCl, 2% SDS, 10% glycerol and 5% β‐mercaptoethanol), and the mixture was boiled for 5 min. Equal amounts (50 μg) of the denatured proteins were loaded onto each lane, separated on 8%‐15% SDS polyacrylamide gels, followed by transfer of the proteins to polyvinylidene difluoride membranes overnight. Membranes were blocked with 0.1% Tween‐20 in PBS containing 5% non‐fat dried milk for 20 min at room temperature, and the membranes were reacted with primary antibodies overnight. The membranes were then incubated with a horseradish peroxidase‐conjugated goat anti‐rabbit or anti‐mouse secondary antibody for 2 h. The blots were detected using an ImageQuant™ LAS 4000 mini (Fujifilm, Tokyo, Japan) with an Enhanced Chemiluminescence substrate (Millipore, Billerica, MA). Densitometry analyses were performed using commercially available quantitative software (AlphaEase, Genetic Technology Inc. Miami, FL), with the control set as 1‐fold, as shown below.

### 2.6 Immunofluorescence assay

A7r5 cells (2 × 10^4^ cells/well) were seeded onto an 8‐well glass Tek chamber and pre‐treated with FKA (2‐30 μM) for 2 h and then stimulated with or without TGF‐β1 (10 ng/mL) for 24 h. After treatment, cells were fixed in 2% paraformaldehyde for 15 min, permeabilized with 0.1% Triton X‐100 for 10 min and then incubated for 1 h with anti‐F‐actin, anti‐Smad3 or anti‐Nrf‐2 primary antibodies in 1.5% FBS. The cells were then incubated with a FITC (fluorescein isothiocyanate)‐conjugated (488 nm) secondary antibody for an additional 1 h in 6% bovine serum albumin. Following this, cells were stained with 1 μg/mL DAPI for 5 min. The stained cells were washed with PBS and visualized using a fluorescence microscope at 200× magnification.

### 2.7 Luciferase activity assay of Smad3 and ARE

The Smad3 and ARE transcriptional activity was measured using a dual‐luciferase reporter assay system (Promega, Madison, WI). A7r5 cells were cultured in 24‐well plates that had reached 70%‐80% confluence and incubated for 5 h with serum‐free DMEM that did not contain antibiotics. The cells were then transfected with either a pcDNA vector or a Smad3 (pGL3‐SBE4‐Luc reporter vector) plasmid/ARE plasmid with β‐galactosidase using Lipofectamine 2000 (Invitrogen, Carlsbad, CA, USA). After plasmid transfection, cells were pre‐treated with FKA 7.5 μM for 0.5 to 4 h and then stimulated with or without TGF‐β1 (10 ng/mL) for 24 h. Following treatment, the cells were lysed, and their luciferase activity was measured using a luminometer (Bio‐Tek instruments Inc, Winooski, VA). The luciferase activity was normalized to the β‐galactosidase activity in cell lysate, which was considered the basal level (100%).

### 2.8 In vitro wound‐healing repair assay

To assess the cell migration, A7r5 cells were seeded into a 12‐well culture dish and grown in DMEM containing 10% FBS to a nearly confluent cell monolayer. The cells were re‐suspended in DMEM medium containing 1% FBS, and a “wound gap” in the monolayers was carefully scratched using a culture insert. Cellular debris was removed by washing with PBS. Then, the cells were incubated with a non‐cytotoxic concentration of FKA (2‐30 μM) for 2 h and stimulated with or without TGF‐β1 (10 ng/mL) for 24 h. The migrated cells were imaged (200× magnification) at 0 and 24 h to monitor the migration of cells into the wounded area, and the closure of the wounded area was calculated.

### 2.9 Cell invasion assay

Invasion assay was performed using BD Matrigel invasion chambers (Bedford, MA, USA). For the assay, 10 μL Matrigel (25 mg/50 mL) was applied to 8‐μm polycarbonate membrane filters, and 1 × 10^5^ cells were seeded to the Matrigel‐coated filters in 200 μL of serum‐free medium containing FKA (2‐30 μM) and/or TGF‐β1 (10 ng/mL). The bottom chamber of the apparatus contained 750 μL of complete growth medium. Cells were allowed to migrate for 24 h at 37°C. After 24 h incubation, the non‐migrated cells on the top surface of the membrane were removed with a cotton swab. The migrated cells on the bottom side of the membrane were fixed in cold 75% methanol for 15 min and washed 3 times with PBS. The cells were stained with Giemsa stain solution and then de‐stained with PBS. Images were obtained using an optical microscope (200 ×  magnification), and invading cells were quantified by manual counting. The inhibition of invading cells was quantified and expressed on the basis of untreated control cells, which were set as 1‐fold.

### 2.10 Measurement of intracellular ROS generation

The accumulation of intracellular ROS in A7r5 cells was quantified by a fluorescence spectrophotometer using DCFH_2_‐DA as described previously.^30^ Briefly, A7r5 cells at a density of 4 × 10^5^ cells/well in 12‐well plates were pre‐treated with FKA (7.5 μM) for 2 h in the presence or absence of TGF‐β1 (10 ng/mL) stimulation for 30 min. Then, the non‐fluorescent probe, DCFH_2_‐DA (10 μM), was added to the culture medium, and the cells were incubated at 37°C for another 30 min. After incubation, the cells were washed with warm PBS, and the ROS production was measured by changes in fluorescence due to the intracellular production of DCF caused by the oxidation of DCFH_2_. The DCF fluorescence was measured via fluorescence microscopy (200 ×  magnification) (Olympus, Center Valley, PA, USA). The fold‐increase in ROS generation was compared with the vehicle‐treated cells, which were arbitrarily set as 1.

### 2.11 Transient transfection of siRNA targeting Nrf2

A7r5 cells were transfected with Nrf2 siRNA using Lipofectamine RNAiMAX (Invitrogen, Grand Island, NY, USA) according to the manufacturer's instructions. For the transfection, cells were grown in DMEM containing 10% FBS and plated in 6‐well plates to 60% confluence at the time of transfection. The next day, the culture medium was replaced with 50 μL of Opti‐MEM, and the cells were transfected using the RNAiMAX transfection reagent. For each transfection, 5 μL RNAiMAX was mixed with 250 μL of Opti‐MEM and incubated for 5 min at room temperature. In a separate tube, siRNA (100 pM, for a final concentration of 100 nM in 1 mL of Opti‐MEM) was added to 250 μL of Opti‐MEM, and the siRNA solution was added to the diluted RNAiMAX reagent. The resulting siRNA/RNAiMAX mixture (500 μL) was incubated for an additional 25 min at room temperature to allow complex formation. Subsequently, the solution was added to the cells in the 6‐well plates, for a final transfection volume of 1 mL. After incubation for 6 h, the transfection medium was replaced with 2 mL of standard growth medium, and the cells were cultured at 37°C. Cells were co‐incubated with FKA (7.5 μM) for 2 h in the presence or absence of TGF‐β1 (10 ng/mL) according for the indicated times, and changes in the protein expressions of Nrf2 (0.5 h), HO‐1 (8 h), NQO‐1 (8 h) and γ‐GCLC (8 h) were determined by Western blotting.

### 2.12 Statistical analyses

The results are presented as the mean ± standard deviation (mean ± SD). The obtained values in this study were analysed using analysis of variance followed by Dunnett's test for pair‐wise comparison. The results are significant at *P <* *0.05, **P <* *0.01 and ***P <* *0.001 compared to control cells and significant at ^#^ *P *<* *0.05, ^##^ *P *<* *0.01 and ^###^ *P *<* *0.001 compared to TGF‐β1‐treated cells.

## 3 RESULTS

### 3.1 Effects of FKA on the viability of SMC (A7r5) cells with or without TGF‐β1‐stimulation

**Figure 1:**
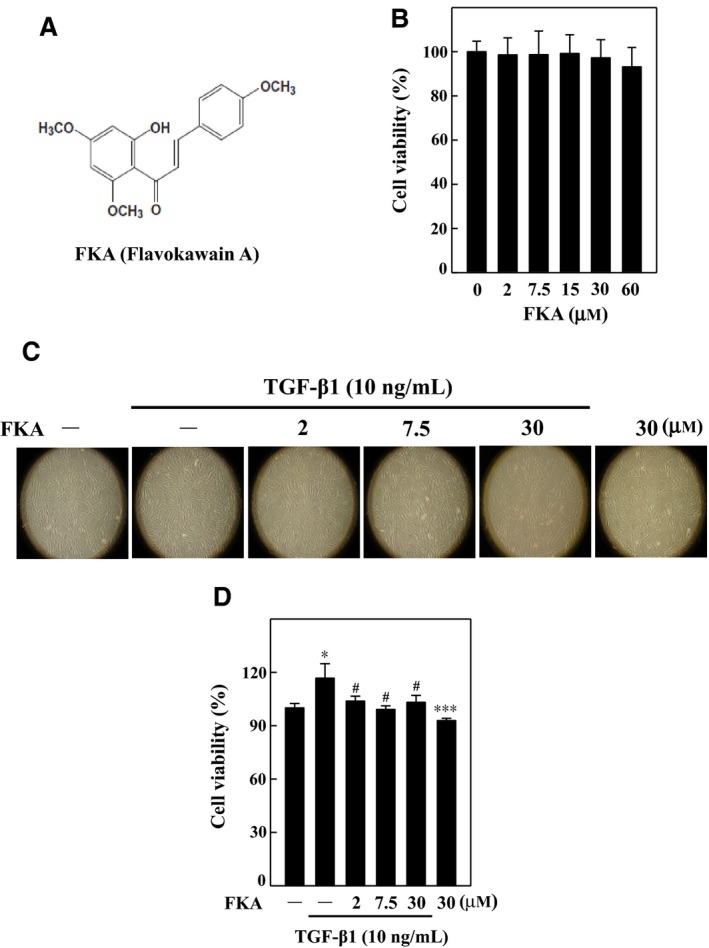
Effects of flavokawain A (FKA) on the viability of vascular smooth muscle (A7r5) cells with or without TGF‐β1 stimulation. A, Chemical structure of FKA. B, Cells were treated with increasing concentrations of FKA (2‐60 μM) for 48 h, and then, cell viability was determined by MTT assay. Cell viability (%) was calculated as follows: (A570 of treated cells/A570 of untreated cells) × 100. C‐D, Cells were pre‐treated with FKA (2, 7.5, or 30 μM for 2 h) and then stimulated with or without TGF‐β1 (10 ng/mL) for 24 h. C, Morphological changes of A7r5 cells were examined by phase‐contrast microscopy (200× magnification). D, The effects of FKA along with TGF‐β1 on cell viability of A7r5 cells were determined by MTT assay. The results are presented as the mean ± SD of three assays. Significant at *P <* *0.05 and ***P <* *0.001 compared to control cells; significant at #*P *<* *0.05 compared to TGF‐β1‐treated cells

FKA and its cytotoxicity on human SMC (A7r5) cells were determined by treating cells with increasing concentrations of FKA (0‐60 μM) for 24 h (Figure 1A). MTT assay results showed that the viability of A7r5 cells remains the same (100%) even after FKA treatment, which indicates that FKA up to 60 μM did not produce any cytotoxic effects (Figure 1B). We further evaluated the effects of FKA (0‐30 μM) on morphology, and cell viability of A7r5 cells was determined in the presence or absence of TGF‐β1 (10 ng/mL) induction. Images from optical microscopy showed no morphological changes following FKA treatment (Figure 1C). FKA treatment (0‐30 μM) significantly suppressed the TGF‐β1 (10 ng/mL)‐induced increased viability of A7r5 cells (Figure 1D). These results indicated that FKA is not cytotoxic for A7r5 cells, despite preventing the excessive growth induced by TGF‐β1.

### 3.2 FKA suppresses TGF‐β1‐induced fibrosis via inhibition of F‐actin, α‐SMA and fibronectin in A7r5 cells

**Figure 2:**
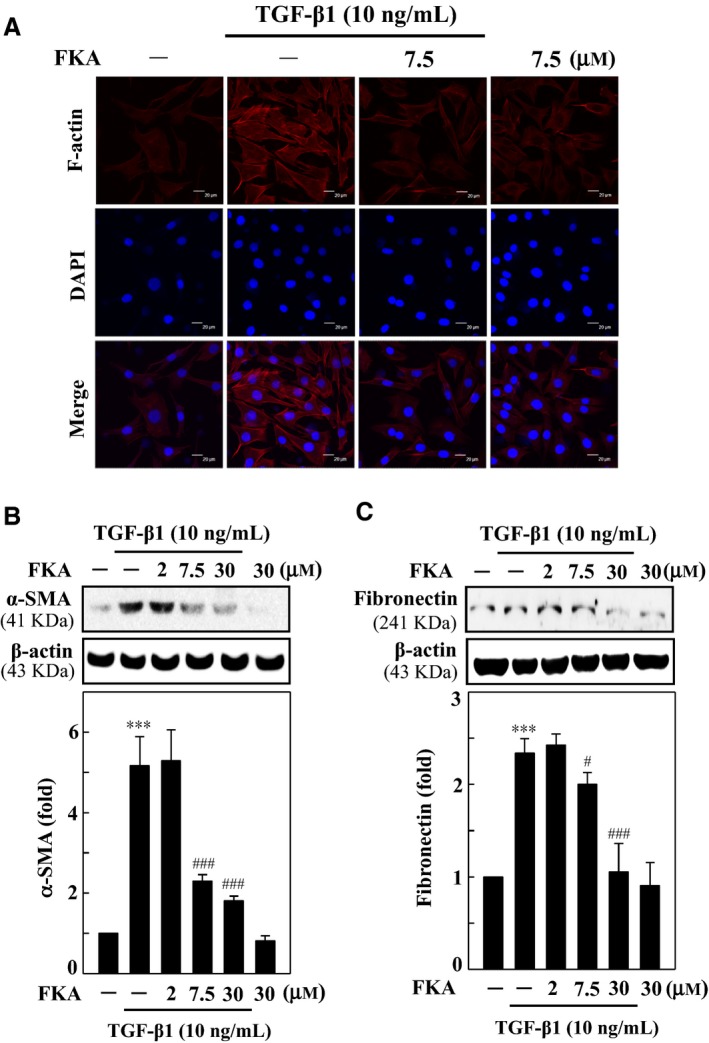
FKA blocks fibrosis through down‐regulation of F‐actin, α‐SMA and fibronectin in TGF‐β1‐stimulated A7r5 cells. (A) Immunofluorescence analysis showed F‐actin distribution in FKA pre‐treated TGF‐β1‐ stimulated cells. Cells were treated with FKA (7.5 μM) for 2 h prior to TGF‐β1 (10 ng/mL) stimulation for 24 h. Cells were stained with DAPI (1 μg/mL) for 5 min and then examined by fluorescence microscopy (200× magnification). Changes in α‐SMA (B) and fibronectin (C) protein levels were monitored by Western blotting. Cells were pre‐treated with FKA (2‐30 μM) for 2 h and then stimulated with or without TGF‐β1 (10 ng/mL) for 24 h. Relative changes in the protein levels were quantified using AlphaEaseFc 4.0 software and densitometric analysis, with the control set as onefold. The results are presented as the mean ± SD of three assays. Significant at ***P <* *0.001 compared to control cells; significant at #*P *<* *0.05 and ###*P *<* *0.001 compared to TGF‐β1‐treated cells

TGF‐β1‐induced vascular fibrosis is characterized by cytoskeletal rearrangements and alterations in F‐actin assembly.^7^, ^8^ To determine TGF‐β1‐induced fibroblastic‐type cytoskeletal rearrangements, we detected the F‐actin structure and appearance in A7r5 cells by immunofluorescence staining. Cells following TGF‐β1 treatment (10 ng/mL, 24 h) showed much thicker central stress fibers that were mostly oriented in parallel to the long axis of the cells (Figure 2A). Interestingly, the abnormal F‐actin structure (stress fiber disruption) was not seen in FKA pre‐treated (7.5 μM) cells (Figure 2A). Control cells without TGF‐β1 stimulation showed randomly oriented cytoplasmic fibers.

TGF‐β1 is known to activate several ECM components, including fibronectin, an essential protein for enhancement of α‐SMA that is involved in fibrosis.^9^ To define the effects of FKA on TGF‐β1‐induced onset of fibrosis, the changes in α‐SMA and fibronectin protein levels were determined. Western blot data showed that the α‐SMA (Figure 2B) and fibronectin (Figure 2C) levels were significantly greater in A7r5 cells following TGF‐β1 treatment (10 ng/mL, 24 h). The fibronectin and α‐SMA levels with TGF‐β1 were increased ~2.5‐fold and ~5‐fold, respectively, when compared with control cells (Figure 2B and C). However, FKA pretreatment (0‐30 μM, 2 h) substantially suppressed both the fibronectin and α‐SMA elevation in a concentration‐dependent manner, with 30 μM FKA more effective than other concentrations.

### 3.3 FKA inhibits nuclear translocation and transcriptional activation of Smad3 in TGF‐β1‐stimulated A7r5 cells

**Figure 3:**
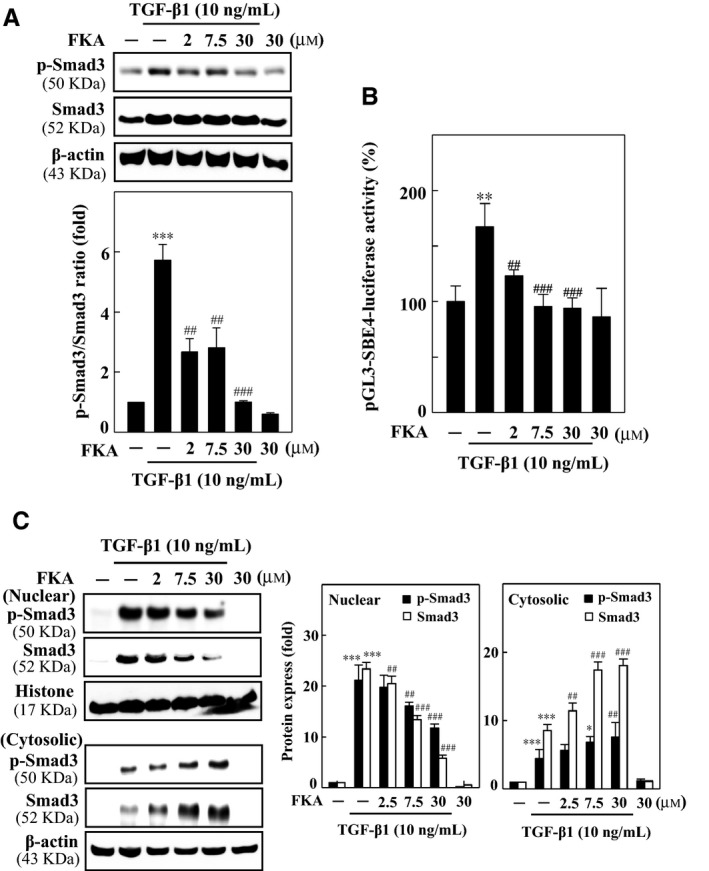
FKA attenuates TGF‐β1‐induced fibrosis through the inhibition of Smad3 in A7r5 cells. For all experiments, cells were pre‐treated with FKA (2‐30 μM) for 2 h and then stimulated with TGF‐β1 (10 ng/mL) for 24 h. A, FKA suppresses the induction of p‐Smad3 levels in TGF‐β1‐stimulated cells. The phosphorylated Smad3 (p‐Smad3) and total Smad3 protein levels were evaluated by Western blotting. B, Transcriptional activity of Smad3 (pGL3‐SBE4‐Luc) was monitored by luciferase reporter assay. Following plasmid transfection, cells were pre‐treated with FKA and then stimulated with or without TGF‐β1. Luciferase activity was determined and normalized to β‐gal activity and shown as relative luciferase activity. C, p‐ Smad3 and total Smad3 protein levels were determined in nuclear and cytosolic fractions of cells. Histone and β‐actin were used as controls. Relative changes in protein intensities were quantified using AlphaEaseFc 4.0 software and presented as a histogram, with a control set to onefold. Significant at **P <* *0.01 and ***P <* *0.001 compared to control cells; significant at ##*P *<* *0.01 and ###*P *<* *0.001 compared to TGF‐β1‐ treated cells

TGF‐β1 increases the phosphorylation of various Smads, particularly Smad3, which forms a heterodimer complex with Smad4 and then translocates into the nucleus to induce fibrotic genes.^13^, ^14^ To provide molecular evidence for the anti‐fibrotic properties of FKA, we evaluated the Smad3 signaling in TGF‐β1‐stimulated A7r5 cells. Cells were treated with FKA (0‐30 μM, 2 h) prior to TGF‐β1 stimulation (10 ng/mL), and then, changes in phosphorylated Smad3 and total Smad3 levels were determined. We found that TGF‐β1 induced an enormous increase in p‐Smad3 levels (>5‐fold) in whole cell lysates, which was dose‐dependently abolished by FKA pretreatment (Figure 3A). Next, we assayed the Smad3 transcriptional activity with a luciferase reporter construct that was stably transfected into A7r5 cells. The results from the luciferase reporter assay revealed that TGF‐β1 (10 ng/mL) stimulation profoundly increased the Smad3 (pGL3‐SBE4) transcriptional activity. Consistent with the decreased p‐Smad3 levels, FKA (0‐30 μM) pretreatment significantly inhibited the Smad3 transcriptional activity in a dose‐dependent manner against TGF‐β1 stimulation (Figure 3B). Correspondingly, increased nuclear accumulation of p‐Smad3 and Smad3 with TGF‐β1 was substantially suppressed by FKA pretreatment (Figure 3C). FKA‐mediated inhibition of p‐Smad3/Smad3 nuclear localization simultaneously increased Smad3 levels in the cytosol. Nevertheless, increased Smad3 in the cytosol with FKA treatment was greater than the p‐Smad3 up‐regulation (Figure 3C). These results showed that FKA can inhibit the TGF‐β1‐activated Smad3 signaling to avert the onset of vascular fibrosis.

### 3.4 FKA pretreatment inhibits TGF‐β1‐induced migration and invasion of A7r5 cells

**Figure 4:**
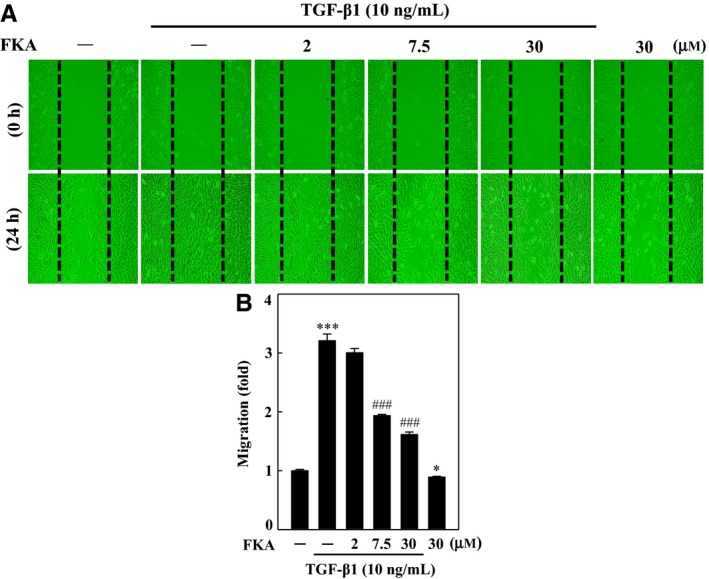
FKA inhibits TGF‐β1‐induced migration. A‐B, Cells were pre‐treated with FKA (2‐30 μM) or vehicle control (0.1% DMSO) for 2 h, and then, migration of cells was determined following 24 h treatment with or without TGF‐β1 (10 ng/mL). A, Cells that migrated to the lower membrane were photographed (200 ×  magnification). B, The percentage of migrated cells was quantified and expressed relative to untreated cells (control), which were set at onefold. To quantify migration, cells were counted in three microscopic fields per sample. The results are presented as the mean ± SD of three assays. Significant at ***P <* *0.001 compared to control cells, significant at #*P *<* *0.05; ##*P *<* *0.01; ###*P *<* *0.001 compared to TGF‐β1‐treated cells

TGF‐β1 is extensively involved in the development of fibrosis in various organs by disturbing the homeostatic microenvironment and promoting cell migration, invasion or hyperplastic changes.^15^, ^16^ We hypothesized that anti‐fibrotic properties of FKA were possibly associated with decreased cell migration and invasion against TGF‐β1 stimulation. The anti‐migration effect of FKA against TGF‐β1 activation was determined by performing a classical wound‐healing assay. In this assay, we found that 24‐ hour TGF‐β1 treatment induced significant migration of A7r5 cells (>3‐fold), which was effectively inhibited by FKA treatment (2 h), particularily at 7.5 and 30 μM doses (Figure 4A and B). We found that TGF‐β1‐induced substantial increase in A7r5 cell migration (>3‐fold), which was significantly inhibited by FKA pretreatment, particularly at 7.5 and 30 μM doses (Figure 4A and B).

**Figure 5:**
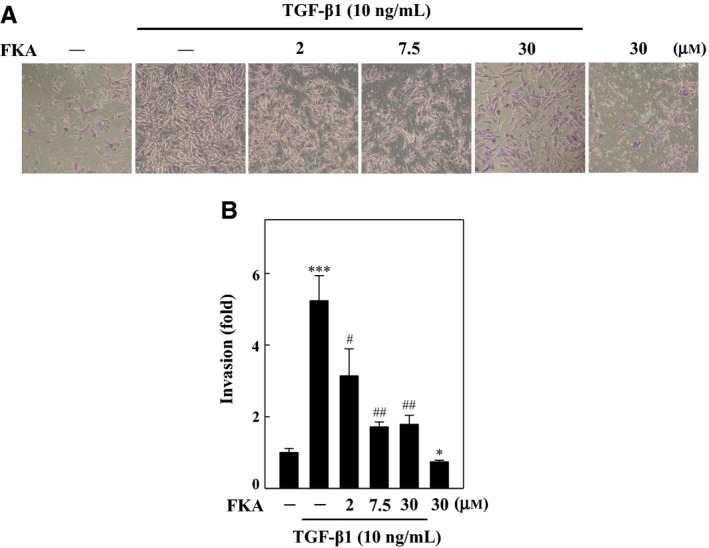
FKA inhibits TGF‐β1‐induced invasion of A7r5 cells. (A‐B) Cells were pretreated with FKA (2‐30 μM) or vehicle control (0.1% DMSO) for 2 h, and then, invasion of cells was determined following 24 h treatment with or without TGF‐β1 (10 ng/mL). A, Cell invasion under the membrane was photographed (200 ×  magnification). B, The inhibition of invading cells was quantified and expressed on the basis of untreated cells (control) that represented onefold. The results are presented as the mean ± SD of three assays. Significant at ***P <* *0.001 compared to control cells; significant at #*P *<* *0.05, ##*P *<* *0.01 and ###*P *<* *0.001 compared to TGF‐β1‐treated cells

We further determined, via transwell invasion assay, the ability of cells to pass through a layer of ECM on a Matrigel‐coated filter in FKA pre‐treated (0‐30 μM) cells following TGF‐β1 stimulation (10 ng/mL). Figure 5A and B show the imaged and quantified invading cells following treatments. Similar to migration, TGF‐β1 profoundly increased the invasion ability of A7r5 cells, by approximately 5‐fold. Interestingly, FKA treatment substantially restrained the TGF‐β1‐induced invasion ability in a dose‐dependent manner. Although FKA exhibited anti‐migration and anti‐invasion properties in the presence of TGF‐β1, FKA alone did not affect the endogenous migration and invasion potential of A7r5 cells.

### 3.5 FKA down‐regulates MMP‐9/‐2 and up‐regulates TIMP‐1 expressions in TGF‐β1‐activated A7r5 cells

**Figure 6:**
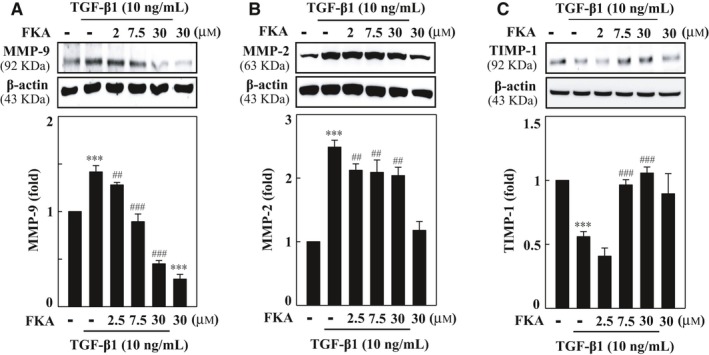
FKA down‐regulates MMP‐9/‐2 and up‐regulates TIMP‐1 expressions in TGF‐β1‐activated A7r5 cells. A‐C, Cells were preincubated with FKA (2‐30 μM) for 2 h and then stimulated with or without TGF‐β1 (10 ng/mL) for 24 h. Western blotting was performed to analyse (A) MMP‐9, (B) MMP‐2 and (C) TIMP‐1 protein levels. Relative changes in protein intensities were quantified using AlphaEaseFc 4.0 software and presented as histograms, with controls set as onefold. The results are presented as the mean ± SD of three assays. Significant at ***p <* *0.001 compared to control cells; significant at ##*p *<* *0.01 and ###*p *<* *0.001 compared to TGF‐β1‐treated cells

MMPs represent a family of proteolytic enzymes that degrades ECM components and plays an important role in cell migration and invasion. TIMPs reduce excessive ECM degradation, and an imbalance between MMPs and TIMPs underlies the pathogenesis of vascular fibrosis.^17^ Based on the anti‐migration and anti‐invasion properties of FKA, we determined the effects of FKA on MMP‐9, MMP‐2 and TIMP‐1 expression levels in TGF‐β1‐stimulated SMCs. Western blot results showed that TGF‐β1 stimulation (10 ng/mL) for 24 h significantly up‐regulated MMP‐9 and MMP‐2 expressions, while it down‐regulated TIMP‐1 levels in A7r5 cells, which facilitated migration/invasion (Figure 6A‐C). Intriguingly, FKA pretreatment (0‐30 μM) remarkably suppressed the TGF‐β1‐induced MMP‐9 and MMP‐2 elevation (Figure 6A and B). Furthermore, inhibition of MMP activation with FKA was accompanied by a significant restoration of TIMP‐1 levels in TGF‐β1‐treated cells (Figure 6C). These results demonstrate that FKA is able to inhibit TGF‐β1‐induced vascular cell migration and invasion, possibly through the inhibition of MMP‐9/‐2 activation and restoration of TIMP‐1 degradation.

### 3.6 FKA diminishes TGF‐β1‐induced intracellular ROS production in A7r5 cells

**Figure 7:**
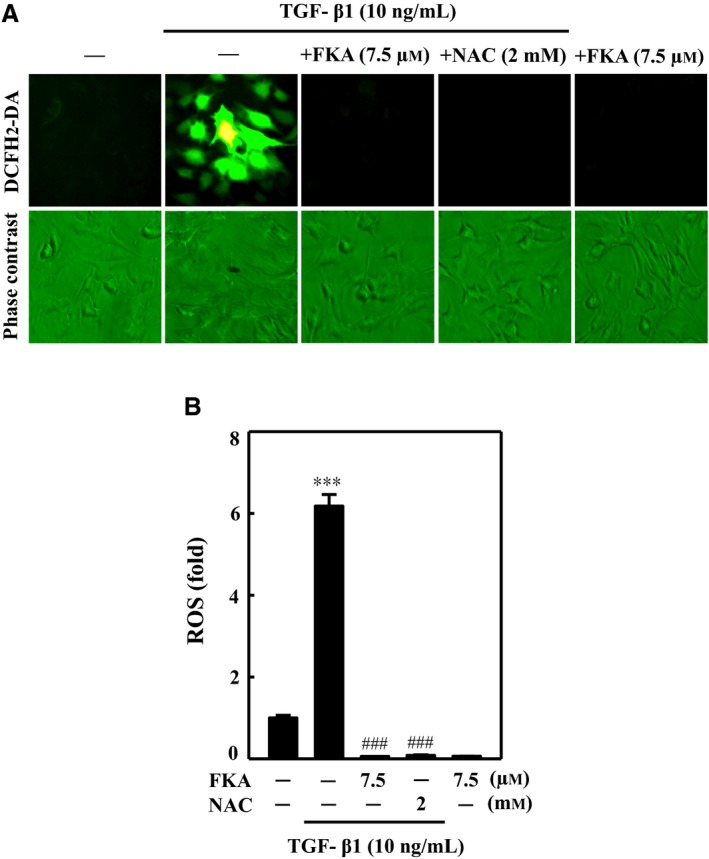
FKA suppresses TGF‐β1‐induced intracellular ROS production in A7r5 cells. A‐B, Cells were preincubated with or without FKA (7.5 μM) or NAC (2 mM) for 2 h and then stimulated with TGF‐β1 (10 ng/mL) for 30 min. A, Intracellular ROS levels were indicated by DCF fluorescence and measured by fluorescence microscopy (200× magnification). B, The percentage of fluorescence intensity of DCF‐stained cells was quantified by Olympus Soft Image Solution software. The percentage of fluorescence intensity (ROS) was compared with that of control cells, which were arbitrarily assigned a value of one. The results are presented as the mean ± SD of three assays. Significant at ***P <* *0.001 compared to control; significant at ###*P *<* *0.001 compared to TGF‐β1‐treated cells

It has been well‐documented that increased ROS production is interlinked with TGF‐β1 production and signaling, which may synergistically participate in the onset of fibrotic events.^2^ Since ROS are key instigators in the pathophysiology of fibrosis, we determined whether FKA is able to inhibit the TGF‐β1‐induced ROS production in A7r5 cells. We found that TGF‐β1 treatment (10 ng/mL) alone enormously increased (~6‐fold) the intracellular ROS production, which was represented by increased DCF fluorescence (Figure 7A and B). Nevertheless, FKA pretreatment (7.5 μM) resulted in decreased fluorescence intensity in A7r5 cells, indicating diminished TGF‐β1‐induced ROS production (Figure 7A). Decreased ROS production with FKA was similar to the ROS levels observed in NAC pre‐treated TGF‐β1‐stimulated cells. This evidence shows that FKA potently suppressed ROS production in a similar fashion to NAC. The ROS scavenging activity of FKA may be involved in the inhibition of TGF‐β1‐induced ROS‐mediated fibrotic pathology.

### 3.7 Inhibition of ROS production by FKA obliterates nuclear localization of Smad3 in TGF‐β1‐activated A7r5 cells

**Figure 8:**
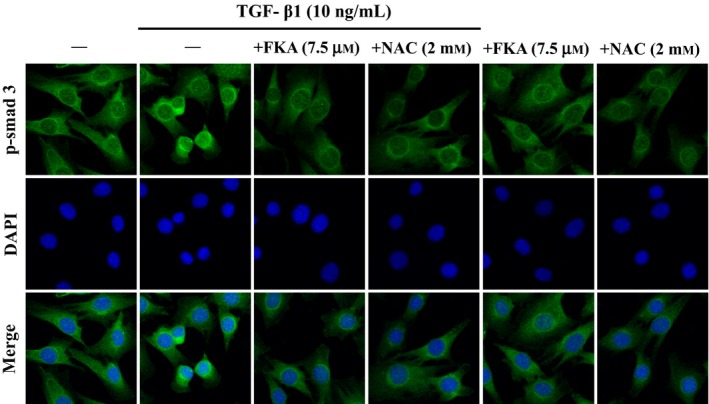
FKA impairs nuclear localization of Smad3 in TGF‐β1‐stimulated A7r5 cells. Cells were pre‐treated with FKA (7.5 μM) or NAC (2 mM) for 2 h and then stimulated with or without TGF‐β1 (10 ng/mL) for 24 h. Immunofluorescence staining was performed to detect the nuclear localization of Smad3. Following incubation with primary antibody (anti‐Smad3) and conjugated secondary antibody, cells were stained with DAPI (1 μg/mL) for 5 min. The sub‐cellular localization of Smad3 in all conditions was visualized under fluorescence microscopy (200 ×  magnification). The results are presented as the mean ± SD of three assays

To further explore the mechanistic role of ROS in the anti‐fibrotic effects of FKA, we determined the nuclear localization of p‐Smad3 in ROS‐quenched conditions (FKA/NAC pretreatment) following TGF‐β1 stimulation. A7r5 cells were treated with FKA (7.5 μM) or an ROS inhibitor (NAC, 2 mM) for 2 h prior to TGF‐β1 stimulation (10 ng/mL), and then immunofluorescence staining was performed to confirm the nuclear import of p‐Smad3. Consistent with Western blot data (Figure 3), images from immunofluorescence assay showed that the TGF‐β1‐induced nuclear accumulation of p‐Smad3 was obliterated by FKA as well as NAC pretreatment (Figure 8). Nuclear localization of p‐Smad3 in FKA treated cells was similar with that of NAC pre‐treated cells. However, Smad3 was restrained in the nucleus of control cells (Figure 8). These findings affirmed that the ROS‐quenching ability of FKA contributed to the inhibition of TGF‐β1/Smad3 signaling in human vascular smooth muscle cells.

### 3.8 Inhibition of ROS production by FKA diminishes TGF‐β1‐induced wound healing migration in A7r5 cells

**Figure 9:**
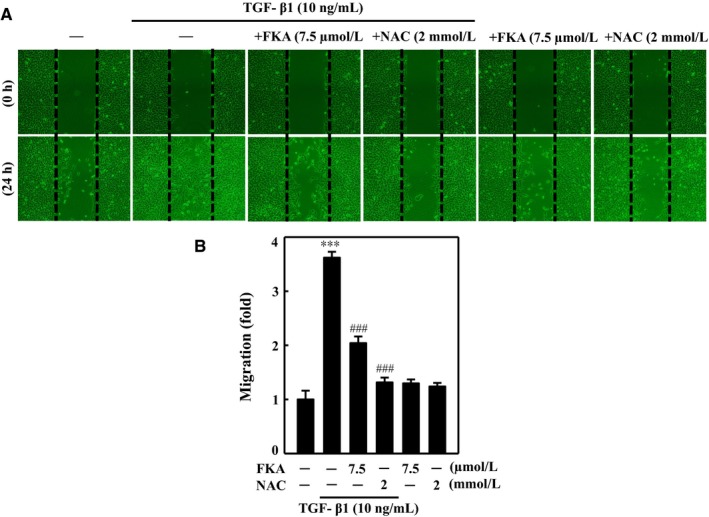
Inhibition of ROS production by FKA diminishes TGF‐β1‐induced wound healing migration in A7r5 cells. Cells were pre‐incubated with or without FKA (7.5 μM) or NAC (2 mM) for 2 h and then stimulated with TGF‐β1 (10 ng/mL) for 24 h. A, Cells that migrated to the lower membrane were photographed (200× magnification). B, The percentage of migrated cells was quantified and expressed relative to untreated cells (control), which were set at onefold. To quantify migration, cells were counted in three microscopic fields per sample. The results are presented as the mean ± SD of three assays. Significant at *P <* *0.05 and **P <* *0.01 compared to control cells; significant at ###*P *<* *0.001 compared to TGF‐β1‐treated cells

It has been described that ROS signaling is involved in TGF‐β1‐mediated fibrosis through activation of proliferation, migration and/or ECM deposition.^2^ Owing to its potent ROS scavenging activity, we speculate that FKA may diminish ROS‐activated migration in TGF‐β1‐stimulated A7r5 cells. To validate the anti‐migration property of FKA, a typical wound‐healing assay was performed, and migration ability was compared with NAC pretreatment. For this assay, confluent monolayers of A7r5 cells were incubated with FKA (7.5 μM) or NAC (2 mM) for 2 h in the presence or absence of TGF‐β1 (10 ng/mL) stimulation for 24 h. We found that TGF‐β1‐stimulated migration (~4‐fold) was substantially inhibited by both FKA and NAC pretreatment. This anti‐migration effect was visualized by noting the widely opened wound areas in FKA or NAC pre‐treated cells, even after 24 h (Figure 9A and B). Since ROS signaling is required for TGF‐β1‐induced migration, suppression of ROS production either by FKA or NAC could contribute to the decreased migration ability of A7r5 cells with TGF‐β1 stimulation.

### 3.9 FKA potentiates Nrf2 activation and nuclear translocation in A7r5 cells

**Figure 10:**
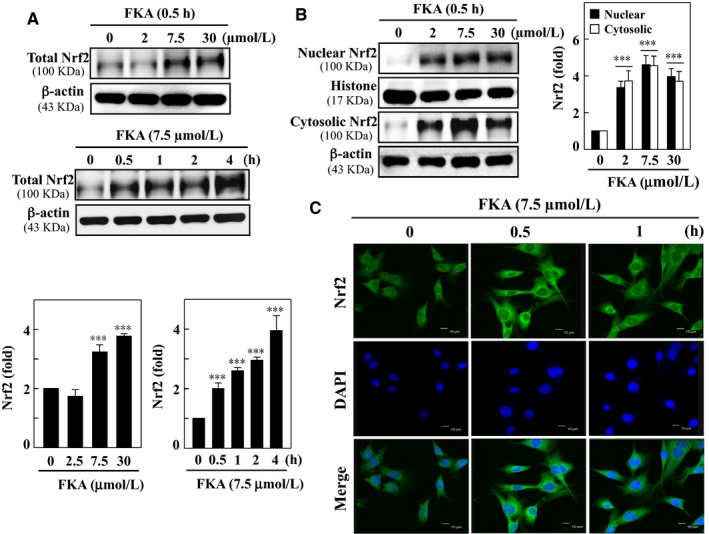
FKA promotes nuclear translocation of Nrf2 in A7r5 cells. A, Cells were incubated with different concentrations of FKA (2‐30 μM) for 0.5 h or a single concentration of FKA (7.5 μM) for 0.5‐4 h. Total cell lysate was subjected to Western blotting to monitor the concentration‐dependent and time‐dependent changes in Nrf2. B, Cells were incubated with FKA (2‐30 μM) for 0.5 h, and changes in Nrf2 levels were determined in nuclear and cytosolic fractions of cells. Relative changes in protein intensities were quantified using AlphaEaseFc 4.0 software and presented as a histogram, with the control set at onefold. C, Immunofluorescence staining to detect the Nrf2 nuclear translocation. Cells were exposed to (7.5 μM) for 0.5 or 1 h, fixed and permeabilized. Cells were incubated with anti‐Nrf2 antibody followed by FITC‐labeled secondary antibody and stained with DAPI (1 μg/mL) for 5 min. Then, the sub‐cellular localization of Nrf2 was visualized using a confocal microscope (630× magnification). All results are presented as the mean ± SD of three assays. Significant at ***P <* *0.001 compared to control cells

The ROS quenching ability of natural compounds is accompanied by enhanced antioxidant responses. Nrf2, a redox‐sensitive zipper protein, translocates into the nucleus in the presence of ROS, binds to ARE in the promoter region and transcribes a set of antioxidant genes.^3^ Since Nrf2 signaling is critically involved in fibrosis pathology, we investigated the effects of FKA on Nrf2 activation in vascular smooth muscle cells. First, we found that FKA treatment (0‐30 μM for 0‐4 h) dose‐ and time‐dependently augmented the total Nrf2 levels in A7r5 cells (Figure 10A). Next, we determined the Nrf2 levels in nuclear and cytosolic fractions of cells following FKA treatment (0‐30 μM for 0.5 h). Western blot results showed that FKA incubation dose‐dependently increased Nrf2 levels in both fractions, with a maximal elevation noted with 7.5 μM (Figure 10B). For further confirmation, we treated cells with FKA (7.5 μM, 0.5‐1 h), and nuclear localization of Nrf2 was visualized using confocal microscopy. Immunofluorescence images confirmed that FKA treatment enhanced the nuclear accumulation of Nrf2, as indicated by strong Nrf2‐staining in FKA‐treated cells (Figure 10C).

### 3.10 FKA triggers ARE promoter activity and up‐regulates HO‐1, NQO‐1 and γ‐GCLC expression levels in A7r5 cells

**Figure 11:**
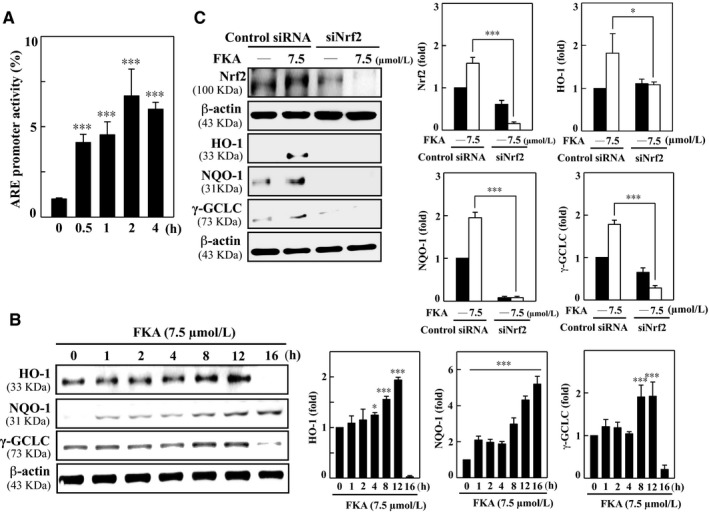
FKA up‐regulates HO‐1, NQO‐1 and γ‐GCLC genes following Nrf2/ARE activation, which is diminished with Nrf2 knockdown in A7r5 cells. A, The luciferase activity of ARE was measured after FKA treatment (7.5 μM) for 0.5‐4 h. Luciferase activity was determined, normalized by β‐gal activity and shown as relative luciferase activity. B, Cells were incubated with FKA (7.5 μM) for 1‐16 h, and total cell lysate was subjected to Western blotting to monitor the changes in HO‐1, NQO‐1 and γ‐GCLC proteins using specific antibodies. C, Cells were transfected with a specific siRNA against Nrf2 or a non‐silencing control. Following transfection (24 h), the cells were incubated with or without FKA (7.5 μM) for the indicated time. Total Nrf‐2 (0.5 h), HO‐1 (8 h), NQO‐1 (8 h) and γ‐GCLC (8 h) were evaluated by Western blotting. The results are presented as the mean ± SD of three assays. Significant at **P <* *0.01 and ***P <* *0.001 compared to control cells

ARE, a *cis*‐acting element, together with Nrf2 induces many antioxidant genes in response to chemical stress. The Nrf2‐ARE transcriptional pathway plays an essential role in the regulation of antioxidant genes and elimination of ROS.^20^ Being a potent Nrf2 activator, we hypothesized that FKA could augment ARE promoter activity and subsequently up‐regulate antioxidant genes in vascular smooth muscle cells. ARE promoter activity was assayed in luciferase reporter co‐transfected cells following FKA treatment (7.5 μM) for 0‐4 h. The results revealed that FKA treatment of A7r5 cells significantly augmented the luciferase activity derived from the ARE promoter (Figure 11A).

Next, to demonstrate the subsequent effects of FKA on endogenous antioxidant genes, we measured the changes in HO‐1, NQO‐1 and γ‐GCLC genes in FKA‐treated (7.5 μM, 0‐16 h) A7r5 cells. Western blot data showed that all antioxidant genes were time‐dependently up‐regulated by FKA treatment. In particular, HO‐1 and γ‐GCLC expression levels were gradually increased until 12 h, whereas NQO‐1 appeared to be up‐regulated until 16 h following treatment (Figure 11B). Based on these findings, we demonstrated that FKA stimulates Nrf2/ARE transcriptional activation to promote antioxidant gene expression in human A7r5 cells.

### 3.11 Nrf2 knockdown diminishes FKA‐induced antioxidant responses in A7r5 cells

Nrf2 signaling is critically involved in the ROS‐mediated regulation of fibrosis; indeed, a lack of Nrf2 expression amplifies oxidative stress and exacerbates fibrotic pathology.^3^ To emphasize the importance of Nrf2 activation for FKA‐induced antioxidant responses, we developed a Nrf2 gene knockdown model by transfecting siRNA into A7r5 cells. Changes in antioxidant gene expression were determined following FKA treatment (7.5 μM). Western blot results showed successful execution of Nrf2 knockdown, as we noted the abolishment of the Nrf2 protein band in siNrf2‐transfected cells even in the presence of FKA (Figure 11C). Consequently, up‐regulated HO‐1, NQO‐1 and γ‐GCLC proteins in control siRNA cells were not observed in Nrf2‐knockdown cells (Figure 11C). These results demonstrate that the FKA‐induced antioxidant response is mediated through Nrf2/ARE signaling in A7r5 cells.

## 4 DISCUSSION

Kava has been used to treat mild to moderate anxiety, insomnia and muscle fatigue in Western countries, which has led to its emergence as one of the ten best‐selling herbal preparations. Several reports of severe hepatotoxicity in kava consumers led the U.S. Food and Drug Administration and authorities in Europe to restrict the sales of kava‐containing products.^31^ The maximum tolerant doses of some chalcones in rodents were shown to be more than 1‐3 g/kg body weight.^29^

FKA exhibited in vivo anti‐tumor activity in a bladder cancer xenograft model and in the UPII‐SV40T transgenic bladder cancer mouse model.^32^ A recent study showed that FKA significantly inhibited LPS‐induced activation of NF‐kB, AP‐1 and JNK/p38 MAPK signaling pathways.^33^ Notably, FKA is more effective than FKB in promoting Nrf2 activation, antioxidant genes (HO‐1 and γ‐GCLC) and GSH levels.^24^ Here, we hypothesized that FKA could alleviate TGF‐β1‐mediated ROS/Smad3 signaling, thereby preventing fibrotic pathology in vascular smooth muscle cells.

TGF‐β1 plays a vital role in tissue remodeling in injured tissues, regulating cell growth and fibrosis. However, aberrant regulation of TGF‐β1 leads to pathologic fibrosis via increased cell proliferation and excessive accumulation of ECM proteins.^34^, ^35^, ^36^ The TGF‐β1 signaling pathway is mediated by a serine/threonine kinase receptor on the cell surface and intracellular Smad proteins. The activated TGF‐β1 receptors propagate the signal through phosphorylation of the Smad proteins. Smad2 and Smad3 participate in TGF‐β1 signals, and Smad4 acts as a common mediator of TGF‐β1.^37^ It has also been well documented that TGF‐β1 increases ROS levels, which mediate the profibrogenic effects of TGF‐β1 via a Smad pathway.^38^ Therefore, inhibition of both ROS/Smad signaling pathways has been suggested as a promising therapeutic strategy for the treatment of vascular fibrosis. Our current results demonstrated that FKA inhibited Smad3 phosphorylation and activation by TGF‐β1, resulting in suppression of fibrogenic gene expression in A7r5 vascular smooth muscle cells. In addition, the increased number of phospho‐Smad3‐immunoreactive cells observed with TGF‐β1 stimulation in A7r5 was significantly decreased by FKA treatment. Thus, at least part of the observed reduction in vascular smooth muscles fibrosis by FKA treatment may be mediated by inhibition of TGF‐β1/Smad signaling. TGF‐β1 stimulates the synthesis of various ECM components, such as fibronectin.^39^ TGF‐β1 activates Smad3, which then associates with the Smad complex and translocates into the nucleus, where it regulates the expression of target genes. In the nucleus, the Smad3/4 or Smad2/3/4 complex can activate transcription by binding directly to a certain consensus sequence, which is present in the promoter region of several TGF‐β1 target genes, including those important in fibrosis, eg, those coding for fibronectin and collagen.^40^ TGF‐β1‐induced Smad3 signaling can stimulate cell migration by up‐regulating fibronectin expression. Fibronectin plays important roles in cell migration, growth and invasion. Several studies have reported that the Smad3 pathway participates in the activation of fibronectin synthesis.^41^

In addition, fibronectin impacts the tensile strength in smooth muscle cells 42 and can increase cytoskeletal organization and mechanical tension generation by cells.^43^ In leiomyomas, mechanotransduction appears to regulate Smad3 activity 44; indeed, in other smooth muscle cells, Smad3 activation correlated with fibronectin polymerization. These data suggest that fibronectin cannot only impact ECM formation but can also regulate intracellular function within the leiomyocyte.^44^ In further support of the role fibronectin plays in the interrelationship between the intracellular structure and ECM, disruption of cytoskeletal actin also disrupts fibronectin matrix organization,^45^ while fibronectin can regulate smooth muscle cell entry into the cell cycle.^46^ Here, we found that the FKA‐induced suppression of fibronectin expression was mediated by inactivation of the Smad3 pathways in fibrosis development. Regulation of fibronectin by FKA provides a mechanism whereby FKA can impact fibrosis, ECM production and intracellular regulation.

Vascular muscle fibrosis is due to both excessive collagen synthesis and abnormal collagen turnover by matrix degrading enzymes, such as matrix metalloproteinases (MMPs).^47^ An important mechanism for the regulation of MMP activities in tissues involves a class of proteins known as tissue inhibitors of metalloproteinases (TIMPs).^48^ TIMPs inhibit MMPs by either binding to the zinc‐binding domain of active MMPs or by binding to the inactive proMMP zymogen, thereby slowing the process of activation.^49^ The MMP and TIMP proteins work in concert to regulate the synthesis and degradation of ECM at wound sites, and the imbalance of MMP and TIMP results in scar formation. Other studies have indicated that TGF‐β1 activates ECM in human glioma.^50^, ^51^ However, the effects of FKA on TGF‐β1‐induced vascular muscle cells are unknown. Here, we report that FKA attenuates migration and invasion in TGF‐β1‐treated vascular cells. More interestingly, cells treated with FKA had reductions in TGF‐β1 induced MMP‐9/‐2 activity via down‐regulation of Smad3. As an early biomarker of fibrosis, TGF‐β1 actuates synthesis of ECM components, such as MMPs. Early examination has shown that chalcone inhibited TGF‐β1‐induced fibrosis on diabetic nephropathy.^52^, ^53^ Here, we found that the chalcone FKA decreased the expression of fibronectin and down‐regulated MMP‐9/‐2 and up‐regulated TIMP‐1 expression in TGF‐β1‐stimulated A7r5 cells, which indicated that FKA might target both TGF‐β1 and its downstream pro‐fibrotic proteins.

TGF‐β1‐induced Smad signaling in ROS is associated with fibrosis progress and development, with differential involvement of Smad3, depending on the cellular context.^54^ In smooth muscle cells, Smad3 signaling has been shown to play a key role in the induction of TGF‐β1‐mediated tissue damage.^55^ Consistent with previous reports, our findings demonstrated the essential role of Smad3 signaling in TGF‐β1‐induced fibrosis in the A7r5 cell line. Our experimental results revealed that FKA pretreatment effectively attenuated the TGF‐β1‐induced Smad3 phosphorylation and transcriptional activity in A7r5 cells. Blocking of TGF‐β1‐induced Smad3 phosphorylation, nuclear accumulation and target gene expression by a specific inhibitor of TGF‐β1 has been found to decrease smooth muscle fibrosis.^56^

Increasing evidence indicates that increasing production of ROS induced by TGF‐β1 plays an important role in fibrogenesis. Reduction of oxidative stress may contribute to inhibition of fibrosis. Nrf2 is a basic leucine‐zipper transcription factor that protects a variety of tissues and cells against oxidative and electrophilic stress through antioxidant response element (ARE)‐mediated induction of diverse phase II detoxification and antioxidant enzymes, including NAD(P)H quinone oxidoreductase 1 (NQO1) and heme oxygenase‐1 (HO‐1), indicating Nrf2‐mediated inhibition of TGF‐β/Smad signaling.^57^ Several studies have shown that Nrf2 activators can potentially inhibit fibrosis, and Nrf2‐null mice were more susceptible to fibrosis than wild‐type mice, which signify that Nrf2 is a potential target for treatment of fibrosis.^58^, ^59^, ^60^, ^61^ Interestingly, many studies have revealed that FKA exhibited anti‐inflammatory and antioxidant activities by activating Nrf2 pathways.^24^, ^31^ In addition, recent reports have also shown that FKA could activate a Nrf2 reporter gene and induce the expression of HO‐1 and NQO‐1.^62^, ^63^, ^64^ However, whether FKA activates Nrf2‐ARE signaling to limit fibrosis has not been previously studied.^65^ Our result suggests that Nrf2 translocated to the nucleus activating ARE reporter gene and inturn inducing the expression of HO‐1, NQO‐1 and γ‐GCLC.

To summarize, we demonstrated that FKA inhibited TGF‐β1 signaling and phosphorylation of Smad3 via upregulation of Nrf2, in turn leading to the activation of antioxidant genes in combating ROS. We elucidate the components of FKA‐mediated inhibition of vascular tissue fibrosis, as well as different underlying sub‐molecular mechanisms that might be investigated in further studies. Unquestionably, elucidating the relative roles of every unique pathway involved in the general effects of FKA in vascular muscle cell protection remains a challenging and important area of study.

## DISCLOSURES

All authors declare that they have no conflicts of interest.
